# GP attitudes to and expectations for providing personal genomic risk information to the public: a qualitative study

**DOI:** 10.3399/bjgpopen18X101633

**Published:** 2019-02-20

**Authors:** Amelia K Smit, Ainsley J Newson, Louise Keogh, Megan Best, Kate Dunlop, Kylie Vuong, Judy Kirk, Phyllis Butow, Lyndal Trevena, Anne E Cust

**Affiliations:** 1 PhD Candidate, Cancer Epidemiology and Prevention Research, Sydney School of Public Health, Faculty of Medicine and Health, The University of Sydney, Sydney, Australia; 2 PhD Candidate, Melanoma Institute Australia, The University of Sydney, Sydney, Australia; 3 PhD Candidate, Sydney Health Ethics, Sydney School of Public Health, Faculty of Medicine and Health, The University of Sydney, Sydney, Australia; 4 Associate Professor, Sydney Health Ethics, Sydney School of Public Health, Faculty of Medicine and Health, The University of Sydney, Sydney, Australia; 5 Associate Professor, Melbourne School of Population and Global Health, The University of Melbourne, Melbourne, Australia; 6 Post-doctoral Fellow, School of Psychology, Psycho-Oncology Co-Operative Research Group (PoCoG), The University of Sydney, Sydney, Australia; 7 Post-doctoral Fellow, Sydney Health Ethics, Sydney School of Public Health, Faculty of Medicine and Health, The University of Sydney, Sydney, Australia; 8 Director, The Centre for Genetics Education, NSW Health, Sydney, Australia; 9 PhD Candidate, Cancer Epidemiology and Prevention Research, Sydney School of Public Health, Faculty of Medicine and Health, The University of Sydney, Sydney, Australia; 10 Senior Lecturer, School of Public Health and Community Medicine, The University of New South Wales, Sydney, Australia; 11 Associate Professor, Westmead Clinical School and Westmead Institute for Medical Research, Faculty of Medicine and Health, The University of Sydney, Sydney, Australia; 12 Professor, Centre for Medical Psychology and Evidence-Based Decision-Making, School of Psychology, The University of Sydney, Sydney, Australia; 13 Professor, Sydney School of Public Health, Faculty of Medicine and Health, The University of Sydney, Sydney, Australia; 14 Associate Professor, Cancer Epidemiology and Prevention Research, Sydney School of Public Health, Faculty of Medicine and Health, The University of Sydney, Sydney, Australia; 15 Associate Professor, Melanoma Institute Australia, The University of Sydney, Sydney, Australia

## Abstract

**Background:**

As part of a pilot randomised controlled trial examining the impact of personal melanoma genomic risk information on behavioural and psychosocial outcomes, GPs were sent a booklet containing their patient’s genomic risk of melanoma.

**Aim:**

Using this booklet as an example of genomic risk information that might be offered on a population-level in the future, this study explored GP attitudes towards communicating genomic risk information and resources needed to support this process.

**Design & setting:**

Semi-structured interviews were conducted with 22 Australian GPs.

**Method:**

The interviews were recorded and transcribed, and data were analysed thematically.

**Results:**

GPs in this sample believed that communicating genomic risk may become a responsibility within primary care and they recommended a shared decisionmaking approach to guide the testing process. Factors were identified that may influence how and when GPs communicate genomic risk information. GPs view genomics-based risk as one of many disease risk factors and feel that this type of information could be applied in practice in the context of overall risk assessment for diseases for which prevention and early detection strategies are available. They believe it is important to ensure that patients understand their genomic risk and do not experience long-term adverse psychological responses. GPs desire clinical practice guidelines that specify recommendations for genomic risk assessment and patient management, point-of-care resources, and risk prediction tools that include genomic and traditional risk factors.

**Conclusion:**

These findings will inform the development of resources for preparing GPs to manage and implement genomic risk information in practice.

## How this fits in

Communicating genomic risk information is anticipated to become a responsibility within primary care but there is currently limited research evidence to guide this process. This qualitative interview study with GPs found that GPs recommend a shared decisionmaking approach to guide the testing process. Factors were also identified that may influence how and when GPs communicate genomic risk information. These findings will inform the development of strategies and resources for preparing the GP workforce to manage and implement genomic risk information in primary care.

## Introduction

Complex diseases such as cancer are often associated with genomic risk factors that are common in the general population. If found to be acceptable, effective, and cost-effective, genomic information could be integrated into disease risk assessment, personalised prevention and risk-stratified screening strategies on a population level.^1,2^ However, high expectations for the future use of genomic risk information (that is, a person’s risk of disease based on common variation across multiple low to moderate penetrance genes) in clinical practice have outpaced translational research on how this new information should be provided,^3,4^ including the role of health professionals such as GPs.

Genetic counselling services are increasingly oversubscribed and may not meet future demand resulting from the expansion of genomics in mainstream health care.^5^ Increasing population awareness of genomics is also leading to greater demand for this type of information, including online personal genomic testing. Patients who undertake personal genomic tests often bring their results to their GP to obtain advice on how to understand and act on this information.^6–8^ These factors are contributing to the anticipation that GPs will be required to apply genomic knowledge to patient care, from disease prevention and early detection to supporting patients through diagnostic and treatment processes.^9,10^

However, systematic reviews show that GPs have limited genomic knowledge and literacy, and low confidence in discussing genomics.^11–13^ These factors are often cited as key barriers to translating genomics into clinical practice^14^ and there has been a recent surge in efforts to prepare GPs for managing genomics in primary care. For example, in 2018 the Royal Australian College of General Practitioners released a resource, Genomics in General Practice, aiming to assist GPs in clinical decisionmaking regarding genomics.^15^ In the UK, there has been ongoing debate about how to prepare GPs for ‘generation genome’^9,16^ and the Royal College of General Practitioners recently released online educational and training resources on genomics for GPs.^17^ Innovative educational programmes and resources aimed at increasing physician knowledge about genomics are also on the rise in the US.^18^ In addition to knowledge and confidence, factors such as attitudes, expectations, and preferences may also influence how GPs communicate genomic risk information in practice.^14^ For example, clinician attitudes towards risk assessment and genetic testing have been found to influence patient decisions about undergoing testing and subsequent clinical management, such as breast cancer risk-reducing surgery.^19–21^ Further, offering genomic testing to the wider population involves a paradigm shift away from traditional genetic testing approaches, where a clinician would order a test when clinically indicated for a particular patient. The context that formed the basis of this study was a more population-based approach to screening and prevention informed by genomic risk information. Understanding GP views on their role in population-level genomic testing will enable better preparation of GPs for the communication of genomic risk information in primary care, but these views remain underexplored.

This research gap was addressed by exploring Australian GP views and attitudes towards communicating genomic risk information and their preferences for tools and resources. An example booklet was used, presenting an individual patient’s personal genomic risk of melanoma as a basis for discussion about GPs potentially being involved in the offer of this type of evidence-based genomic test to the broader (otherwise heathy) population. As GP attitudes are complex and evolving,^14^ qualitative interviews were used, which are recommended for the exploration of understandings, perceptions, and constructions of topics in which participants have a personal stake.^22^ These findings contribute much needed evidence to inform workforce preparation and further resource development for the appropriate expansion of genomics into primary care.

## Method

### GP recruitment

A pilot randomised controlled trial was previously conducted in New South Wales, Australia, in which participants provided a saliva sample for genotyping and received their personal genomic risk of melanoma information in a hardcopy booklet; an educational booklet on melanoma prevention and early detection; and a phone call from a genetic counsellor (key findings have been published elsewhere).^23–25^ In the pilot trial, participants were asked whether they would like to have a copy of their genomic risk booklet sent to their GP, and this was elected by most participants (79%). Participants could also give written consent for the research team to contact their GP and invite them to take part in an interview. Other GPs were recruited via direct contacts or snowball sampling, which involved GPs who had already been interviewed sharing the participant information statement and consent form with other GP colleagues. There were no differences in the conduct of the interviews between GPs recruited via the pilot trial and those recruited via snowball sampling, except that the former were asked to describe any conversations they had with their patient regarding their genomic risk booklet.

### Semi-structured interviews

Interviews were conducted and recorded by a member of the research team in person or via telephone, and were professionally transcribed. GPs were provided with a sample copy of the personal genomic risk of melanoma booklet provided to participants in the pilot trial as a reference point for the interview discussion, but the interview content was broad and included all diseases. The interviews were structured by a discussion guide (further information available from the authors on request), which was developed from the literature^12,14,22^ and with input from the multidisciplinary research team, including GPs. The main topics covered in the interviews included the role of genetics and genomics in current practice, their thoughts on the genomic risk booklet, and their attitudes and expectations towards potentially providing personal genomic risk information (for various diseases, not limited to melanoma), and resources needed to support this process.

### Qualitative analysis

This qualitative study was grounded in a pragmatic paradigm that allows for the combination of diverse sources and methods to conduct objectives-driven research. Methodological pluralism is reflected in the study design as the reference point was a pilot randomised controlled trial and constructivist (qualitative) methodology was used to explore GP knowledge and experiences.^26^ A thorough and systematic thematic analysis approach was used to analyse the interview data.^22,27,28^ This entailed developing a coding framework through reading and re-reading the transcripts and coding the data systematically. An inductive strategy was used to derive themes directly from the data, rather than drawing on pre-selected theoretical concepts or *a prior*i themes to guide the analysis.^22,28^ The themes were developed during the coding process (after interviews had been conducted and transcribed) by reading data across the transcripts and identifying key patterns and ideas that were relevant to the research question.^22,28^ Interviews were conducted until no new themes or sub-codes were identified in the transcript data (data saturation was reached) after five interviews were coded consecutively.^29^ The key themes presented in this study were selected because of their relevance to the research question. The coding was facilitated by NVivo qualitative data analysis software (version 11) and was conducted by two members of the research team, who met to compare coding and discuss discrepancies, which were resolved through consensus. Reflexivity was achieved through regular discussions about codes and themes between the coders and members of the research team, due to their different professional backgrounds.^30^ This allowed discussion of interpretations and competing conclusions from the thematic analysis.^31^ The interviewer, coders, and members of the research team also engaged in discussions about their preconceptions regarding incorporating genomics into population-level prevention and screening strategies, and a range of views (positive and neutral) were engaged with throughout the thematic analysis process to avoid researcher bias. The conduct and reporting of this study follows the Standards for Reporting Qualitative Research.^32^

## Results

### GPs

Fifty-nine GPs were invited via the pilot trial participants, of whom 13 (22%) gave consent and participated in an interview. Nine other GPs who participated were recruited via snowball sampling or via direct contacts. About half of the GPs were female and the mean length of experience in general practice was 12 years. Interviews lasted on average 20 minutes (standard deviation 5 minutes) and most were conducted via telephone.

Below are the key themes that relate to GPs’ experience with genomics-based disease risk information in practice, their attitudes towards potentially providing this type of information to the general population, and their preferences for future tools and resources, including their views on the personal genomic risk of melanoma booklet provided to participants in the pilot trial. No differences in themes or patterns in ideas were observed between GPs recruited via the pilot trial and those recruited via snowball sampling.

### Personal experience with genomics-based disease risk information in current practice

Some GPs perceived genomic risk information to be potentially useful for preventive health care and to inform screening frequencies:

*'But it fits into the general spectrum of health screening and preventative health for us. So if there was any knowledge of gene which put someone at increased risk, then we would certainly put them under closer surveillance *[*…*] ' (GP 1092)

GPs also emphasised that genomic risk should be explained to patients within the context of other disease risk factors:

*'I think that it's important, but I think it needs to be put in its place. But you know, genetics do play a role; 50% of obesity is genetic. But you have to make sure that* […] *they don't start to think it's a foregone conclusion.*' (GP 380)

Discussing genomic risk information with patients was closely linked to conversations about family history of disease, particularly cancer(s). Similarly, GPs’ experience was limited to discussing rare, high penetrance variants associated with familial cancers as opposed to discussing genomic testing to predict the risk of more common, complex traits. Most GPs reported that the frequency of conversations about DNA-based risk was low. Conversations about genomic risk information were generally initiated by the patient:

*'I'm getting more and more enquiries from people with regard to their genetic risk of different sorts of cancers. So breast cancer is obviously the one that people enquire most about, bowel cancer comes up occasionally, and then people with just strong family histories of different sorts of cancers.*' (GP 2003)

GPs also reported that patients with higher levels of education had more interest in and questions about genomics. As a result of increasing public awareness and media attention, some GPs reported that:

*'Doctors are asked to have these conversations* [about genomics] *more and more, and quite possibly in primary care they're not as up to speed with the available science.*' (GP 2001)

GPs reported mixed levels of confidence in discussing genomic risk information with patients. Some felt confident with providing general explanations of genetics in relation to disease risk, particularly if it involved single-gene variants with clear inheritance patterns:

*'On the level that I talk about it, pretty confident, but I'm talking fairly basic levels* […] *I'm fully aware of how complicated the full picture is, but I'm talking, recessive, dominant, sex-linked sort of level of discussion, so I'm pretty confident with that.*' (GP 1224)

But the provision of detailed genomic risk information was considered to be a specialist responsibility:

*'I mean we assess risk in patients, depending on their personal history and family history, but I guess the genetic or the hereditary part of it is often dealt with by sort of specialists who sort of do further testing and then determine their true sort of risk*.' (GP 2005)

### Views on providing personal genomic risk information to the general population

GPs described their expectations towards communicating genomic risk information in practice:

*'… medicine's changing rapidly and this will become or is part of it already and really our genetic clinics are now genetic physicians who provide that information; you can't get into them, they're just so booked out for months. So I do think GPs are going to have to step up and provide that information.*' (GP 748)

GPs felt that they should retain control over the testing process rather than patients undertaking testing via other third party (commercial) providers, but also that patients should be supported to make informed decisions:

*'I wouldn't like patients to be able to ring up the Cancer Council and go, "I want to do one of those melanoma risk tests and get the booklet, take it to my GP" *[…] *I think it would be more effective if it was a conversation where "Okay* [patient name]*, there's this test we can do to assess your risk of melanoma since you're so worried about mum and dad having had it, how would you feel about having the test then we can talk about what your actual risk is?"'* (GP 2001)

Their perceived future role as providers of genomic risk information was complicated by factors such as feeling unprepared and concerned about potential adverse implications. Risk statistics and concepts, and uncertainty were described as difficult for GPs to adequately explain, and for patients to understand, within a single consultation:

*'Risk in general is a very hard thing* […] *So for a busy GP, trying to go through all of this, you know, explaining risk to people is quite a difficult thing.*' (GP 1792)

*'Some people, will want to know everything; other people won't be able to understand the implications of everything either *[…] *There'd be a limit to some people's understanding, even well educated people, they might not fully understand some of the implications.*' (GP 1529)

GPs were concerned about potential adverse implications resulting from receiving personal genomic risk information:

*'You've just got to be careful who that information — who they make that information available to, the applications for insurance, I suppose and whether they're sharing it with family, the implications for the mental health and other family as well.*' (GP 2000)

They felt that patients who may be at risk of adverse psychological responses should be identified early to mitigate this reaction through counselling or discouraging testing:

*'I think it's actually choosing the patient that it will be helpful for, one who just wants some information, isn't overly worried, just needs to, you know, work out her risk level and then do the appropriate thing.*' (GP 1792)

GPs described potential benefits of delivering personal genomic risk information such as motivating health behaviour changes, but these perceived benefits were accompanied by scepticism and uncertainty about the impact on preventive behaviours:

*'I suppose, theoretically, if people did know that they had an increased risk of getting something they would be more motivated to avoid it by putting in lifestyle pattern risk. But people already know if they do this or that, then they’re more likely* [to develop disease.]' (GP 1108)

Many GPs believed that genomic risk information should be limited to diseases for which modifiable prevention strategies are available:

*'If there's something that they can do that's modifiable and it's been explained to them that there's something that they can do that modifies this, then having that information is fine. I think where there's nothing they can do about it, I don't know what sort of extra benefit it has for your health, you know?*' (GP 2000)

### Resources and requirements for the future

The genomic risk booklet developed for the pilot trial was used to provide context for discussing potential resources and requirements needed in the future, and was described as:

*'quite good in terms of the information *[…] *and at the same time, reassuring the patient as well'* (GP 1929)

GPs strongly felt that specific recommendations for practical steps for prevention and early detection behaviours should be provided alongside genomic risk information for patients and clinicians:

*'Where you were mentioning things you could do to minimise your risk* [in the booklet]*, even that was quite vague, I thought, quite non-directive. So I think if it were more specific information, then that would be useful for doctors and for patients.*' (GP 1092)

The value of providing this genomic risk information was dependent on clear implications for clinical practice, and evidence that this information can lead to behaviour change:

*'Look, I think it is useful for patients, it's a matter of what actually you do with it, in terms of what the patient does with it and what their primary care provider does with it as well.*' (GP 748)

Many GPs believed that genetic counselling would be important to assist patients to understand their genomic risk, but felt this would be most appropriate for patients at high disease risk or with complex circumstances:

*'I think the decision to order the test could be a conversation between the patient and the doctor, but then if there was a high risk, probably worthwhile bringing a genetic counsellor in, because then I think people have a lot more questions about "are my children at risk", all those sort of typical genetic questions.*' (GP 2001)

Preferences for tools and resources included risk calculators (similar to those used for assessing cardiovascular disease risk) that incorporate genomic factors alongside traditional disease risk factors and easy to access, evidence-based resources that are regularly updated with changes in evidence and recommendations:

*'If you could incorporate the genetic risk calculator into medical software for melanoma, that would be really powerful and that could be incorporated into part of the automatic stuff that doctors might use when they're performing skin checks on people or preventative healthcare checks on people.*' (GP 2003)

Some GPs believed that future communication of genomic risk information might vary across general practices due to differences in patient populations, such as level of education and socioeconomic status, especially because, as one GP noted:

*'It's when patients come to us asking questions and essentially we start looking for answers, or when we get thrown a letter back after a referral that we don’t know what they're talking about, that promises to actually do something about it.'* (GP 2000)

## Discussion

### Summary

These findings demonstrate that GPs perceived themselves to be future communicators of genomic risk information and supported a patient-centred approach to guide this process. Several considerations have been identified that may influence how and when GPs communicate genomic risk information to patients. GPs view genomics-based risk as one of many disease risk factors and feel that this type of information could be applied in practice in the context of overall risk assessment for diseases for which prevention and early detection strategies are available. They believe it is important to ensure that patients understand their personal genomic risk and do not experience long-term adverse psychological responses. GPs also strongly desire clinical practice guidelines that specify recommendations for genomic risk assessment and patient management, evidence-based point-of-care resources, and risk prediction tools that include genomic and traditional risk factors.

### Strengths and limitations

Over half of the GPs in this study were recruited via participants in the pilot trial and others were recruited via snowball sampling. Therefore, the views that have been explored are likely to be limited to GPs with a specific interest in this topic. This was an exploratory study, and therefore the discussion was broad and covered a range of genomic tests for various diseases. Nevertheless, a wide range of viewpoints were explored that are important for the development of resources from which GPs may benefit in the future. As the reference point for the interviews was an evidence-based genomic test and the corresponding genomic testing results in a booklet, the focus of most discussion was on the communication of these results and potential broader implications (rather than how GPs may decide to offer certain tests to patients). Future research could examine how GPs decide to offer genomic tests based on population-based criteria as opposed to individual-level clinical indications in clinical practice.

### Comparison with existing literature

Overall, GPs in this study viewed genomics and genetics as specialist areas that are highly technical and complex. They attributed this perception to their limited confidence in discussing genomic risk and in explaining complex risk information in general. This view is comparable to studies on GP views towards genetic testing for higher penetrance variants. A study by Puryear *et al* in the US found that GPs described themselves as ‘gatekeepers’ to genetic testing but did not feel confident in this role because it conflicted with their responsibility for maintaining a broad skillset required for primary care.^33^ Other studies have found that GP attitudes depend on how they perceive genetic testing in relation to their primary care responsibilities.^34^ This study found that GPs were supportive of explaining genomic risk information to patients as part of overall risk assessment for preventive care, which they described as a key responsibility. This also depended on the clinical utility of genomic risk information, however.

GPs in this study were concerned about their patients’ ability to interpret genomic risk information and its potential to cause long-term adverse psychological impacts. They felt that easy-to-understand educational information should be provided to patients to help mitigate such impacts, such as the booklet used in the pilot trial. Although this concern about adverse impacts is consistent with other studies on barriers to GP communication about genetics in general practice,^12,33^ most research evidence shows little or no long-term psychological morbidity resulting from undertaking genetic or genomic testing for a range of conditions.^35,36^

Despite their concerns, GPs felt that genetic counselling should be offered only to patients receiving high-risk results as opposed to offering genetic counselling to all patients who undertake genomic testing. Triaging the provision of pre- and post-test genetic counselling according to level of risk or susceptibility to adverse psychological reactions is a potential strategy that is less resource-intensive than current approaches. Biesecker *et al* found that a web-based platform compared to in person consultation was non-inferior in relation to generating knowledge of recessive inheritance, test-related distress, and decisional conflict, and thus proposed reserving in person genetic counselling for health-threatening results.^37^ Ormond *et al* have proposed three levels of communication in the genomic testing process that could be delivered by non-genetics clinicians, depending on patient characteristics and clinical judgment: brief communication (with a clinician), targeted discussion (with the ordering clinician), and traditional genetic counselling (with a clinician with genetics expertise).^38^ The present study's findings suggest that GPs would be willing to adopt such triaged communication approaches for the provision of genomic risk information.

GPs in this study were open to the delivery of genomics in primary care, but felt they were mostly unprepared and had gaps in their knowledge about genomics. Similarly, Chowdury *et al* found that non-genetic health professionals had limited understanding about the contribution of common genetic variations in disease risk and the implications of using genomics in risk-assessment,^39^ but — rather than improving knowledge — systematic reviews suggest that GPs may benefit more from the ability to find relevant genomic information when needed.^13^ GPs in the present study also used the language of shared decisionmaking to describe their future role in communicating about genomic testing with patients. Shared decisionmaking is a process in which clinicians and patients work together to select management options based on clinical preferences and patients’ informed preferences.^40^ This involves the provision of evidence-based information about options, outcomes, and uncertainties to patients, and is considered appropriate for decisions about screening and preventive strategies.^41^ These findings support the need for point-of-care genomic risk assessment and educational resources and tools that can provide shared decision support during consultations and can be updated with current, evidence-based information.^37,42^

### Implications for research and practice

In summary, several factors have been identified that may guide revisions to existing resources and the development of future resources. These include addressing GP concerns about adverse long-term psychological impacts of providing genomic risk information, providing support on optimal methods to explain probabilistic disease risk information, point-of-care tools and resources, and providing education on the relevance of genomic risk information within the broader context of GP responsibilities, for example in preventive care. These findings inform the further development of resources and tools aimed at preparing the GP workforce, addressing information needs and facilitating the appropriate management of genomics in primary care.
